# Diabetic retinopathy predicts cardiovascular mortality in diabetes: a meta-analysis

**DOI:** 10.1186/s12872-020-01763-z

**Published:** 2020-11-04

**Authors:** Xiao-Hong Xu, Bo Sun, Shan Zhong, Dong-Dong Wei, Ze Hong, Ai-Qiang Dong

**Affiliations:** 1 Department of Endocrinology, The Second Affiliated Hospital of Zhejiang University School of Medicine, Hangzhou, Zhejiang, 310009 China; 2 Department of Cardiovascular Surgery, The Second Affiliated Hospital of Zhejiang University School of Medicine, Hangzhou, Zhejiang, 310009 China

**Keywords:** Diabetic retinopathy, Cardiovascular disease, Mortality, Diabetes, Meta-analysis

## Abstract

**Background:**

The prognostic significance of diabetic retinopathy (DR) for cardiovascular diseases (CVD) remained unclear. Therefore, we performed this meta-analysis to assess whether DR predicted CVD mortality in diabetic patients.

**Methods:**

We searched PubMed, Embase, Web of Science and Cochrane Library for cohort studies reporting the association of DR and CVD mortality. Then we pooled the data for analysis.

**Results:**

After screening the literature, 10 eligible studies with 11,239 diabetic subjects were finally included in quantitative synthesis. The pooled risk ratio (RR) of DR, mild DR, and severe DR for CVD mortality was 1.83 (95% confidence interval (CI): 1.42, 2.36; *p* < 0.001), 1.13 (95% CI 0.81, 1.59; *p* = 0.46), and 2.26 (1.31, 3.91; *p* = 0.003), respectively, compared to those without DR. In type 2 DM, the patients with DR had a significantly higher CVD mortality (RR: 1.69; 95% CI 1.27, 2.24; *p* < 0.001). Subgroup analysis also showed a significantly higher CVD mortality in DR according to various regions, study design, data source, and follow-up period (all RR > 1; all *P* values < 0.05). Data from 2 studies showed no significant correlation of DR and CVD mortality in diabetic patients receiving cardiovascular surgery (RR: 2.40; 95% CI 0.63, 9.18; *P* = 0.200).

**Conclusions:**

DR is a risk marker of cardiovascular death, and severe DR predicts a doubled mortality of CVD in diabetes. These findings indicate the importance of early identification and management of diabetic patients with DR to reduce the risk of death.

## Introduction

Diabetes mellitus (DM) has become one of the largest public health challenges throughout the world, both in developed and developing countries [1]. According to the global estimate from the International Diabetes Federation (IDF), there were about 415 million diabetic patients in 2015, and the number will probably rise to 642 million by 2040 [2]. The main burden of DM results from its complications, which can be traditionally divided into macrovascular complications (e.g., cardiovascular disease (CVD)) and microvascular complications (e.g., renal disease, retinopathy, and polyneuropathy) [3].

Diabetic retinopathy (DR) is a common microvascular complication of DM, and has emerged as the leading cause of irreversible blindness in working-age population [4]. DR can be further classified as non-proliferative diabetic retinopathy (NPDR) and proliferative diabetic retinopathy (PDR). In the Wisconsin Epidemiologic Study of Diabetic Retinopathy (WESDR), the overall 10-year incidence of retinopathy in diabetes was 74% [5]. And in patients with retinopathy at baseline, 64% developed more severe retinopathy and 17% progressed to PDR finally [6].

It has been reported that individuals with DM have poorer survival rates than those without, mainly due to the incidence of CVD [7, 8]. Therefore, efforts have been made to clarify the prognostic factors for mortality in diabetes. Although several investigations have found DR is associated with all-cause mortality and incidence of CVD events in both type 1 and type 2 DM [9], it still remains unclear whether DR serves as an indicator of CVD mortality. The inconsistent findings may result from variations in population, study design, sample size, type of DM, duration of diabetes and other factors. Clarifying the relationship between DR and CVD mortality may have significant public health implications for the primary prevention. Therefore, we performed this meta-analysis to assess whether DR is able to predict CVD mortality.

## Methods

### Literature search

The protocol of the meta-analysis was registered in PROSPERO website (University of York, York, UK) with a registration number of CRD42020194324. We searched PubMed, Embase, Cochrane Library, and Web of Science using following keywords with various combinations: “retinopathy”, “diabetic retinopathy”, “DR”, “diabetes”, “diabetes mellitus”, “cardiovascular disease”, “vascular disease”, “coronary heart disease”, “myocardial infarction”, “heart failure”, “heart disease”, “mortality”, and “death”. The last search date was June 1st, 2020 and the literature was limited to human study only.

### Study selection

Inclusion criteria were: (1) prospective or retrospective cohort study based on population or hospital; (2) the study included at least 100 participants; (3) the median follow-up period was more than 2 years; (4) the retinopathy was examined and graded by ophthalmologists using ophthalmoscope or fundus photography, with or without applying fundus fluorescence angiography (FFA); (5) the study reported the CVD mortality in diabetic patients with and without DR; (6) deaths related to CVD included death due to various cardiovascular causes, including myocardial infarction, coronary heart disease, and heart failure; and (7) English-language bibliography.

Exclusion criteria were: (1) studies that based on the same population and failed to provide additional information; (2) ongoing or unpublished studies; and (3) unavailable to obtain the original data. The literature was independently screened and selected by two researchers (XHX, AQD), and any disagreements between them were resolved by discussion.

### Quality assessment and data extraction

For the articles that passed the primary screening, they were reviewed by two authors (XHX, AQD). They independently evaluated the quality of the studies according to the STROBE statement [10]. The research with low quality or evident defects in study design were excluded from this meta-analysis. Any disagreements between the two reviewers were resolved through discussion or judged by senior researchers. The extracted data were: (1) basic characteristics of the included studies and participants; (2) the CVD mortality in diabetic patients with and without DR.

### Statistical analysis

The quantitative synthesis was performed using RevMan 5.3 software (Cochrane Collaboration, Denmark). The risk ratio (RR) with 95% confidence interval (CI) was calculated for dichotomous variables. Forest plots were used for presenting the RR for CVD mortality. In this study, DR was further classified as mild and severe DR. Mild DR was defined as mild to moderate NPDR. Severe DR was defined as severe NPDR, PDR and vison-threatening diabetic retinopathy (VTDR). Subgroup analysis was performed according to the type of DM, regions, study design, data source, follow-up period and surgery condition. The statistical heterogeneity among studies was analyzed using the chi-squared test and presented as the I-squared (less than 50%: low heterogeneity, 50% to 75%: moderate heterogeneity, and more than 75%: high heterogeneity). Fixed-effects model was applied when the heterogeneity was lower than 50%, otherwise, random-effects model was used. The publication bias for included studies was evaluated using Funnel plots. Two-sided *P*-value lower than 0.05 was considered as statistically significance.

## Results

**Fig. 1:**
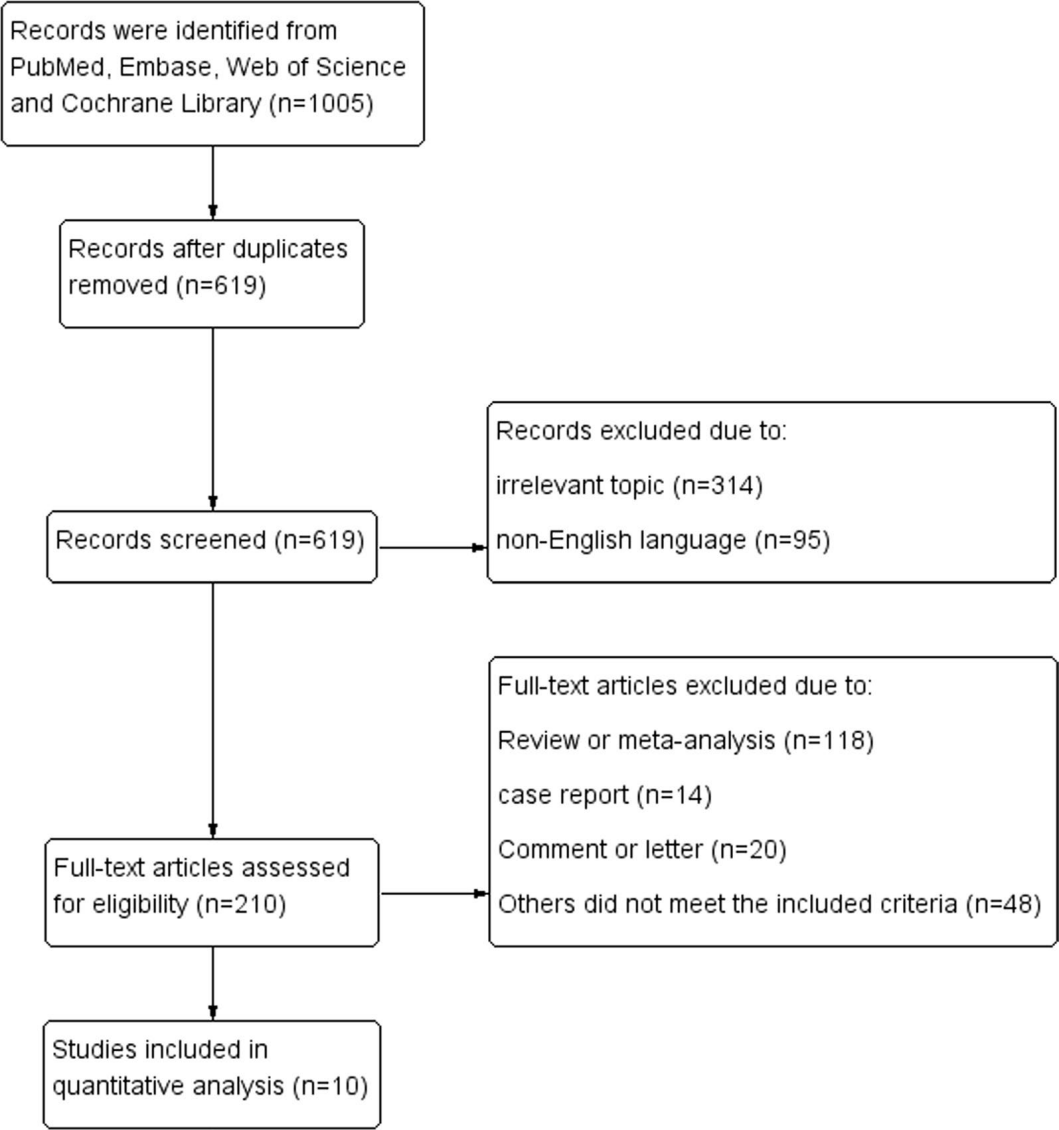
Flow diagram of literature selection

Figure 1 showed the process of the literature selection. At the initial searches, a total of 1,005 articles were potentially eligible (319 from PubMed, 453 from EMBASE, 2 from Cochrane Library, and 231 from Web of Science). After primary screening and removing duplicates, 210 potentially eligible articles were selected. After full-text review, 10 eligible cohort studies with 11,239 diabetic patients were finally included in the quantitative synthesis [11–20].

The included cohort studies consisted of 7 prospective studies and 3 retrospective studies. 7 of them were based on hospital and others were based on population. Data from 5 studies were based on Asian countries and others were based on the western countries. Most participants in the included studies were type 2 DM (T2DM), and subjects in 2 studies underwent percutaneous coronary intervention (PCI) [13] or coronary artery bypass graft (CABG) surgery [20] for the treatment of coronary heart disease (Table 1).

**Table 1:** Basic characteristics of the included studies

First author	Year	Country	Study design	Data source	Follow-up (y)	Sample size	Type of DM	Male (%)	Age at baseline (y)	Diagnosis of DR
Sabanayagam [11]	2019	Singapore	Prospective cohort study	Population-based	Median 8.8	2964	T2DM: 98.2%	50.6	40–80	Fundus photography
Takao [12]	2020	Japan	Retrospective cohort study	Hospital-based	Median 18.6	1902	T2DM	80.3	Mean 55.6	Ophthalmoscope FFA
Kim [13]	2002	Korea	Prospective cohort study	Hospital-based	2	365	T2DM	NA	NA	Ophthalmoscope
Rajala [14]	2000	Finland	Prospective cohort study	Hospital-based	4	428	T2DM: majority	31.8	27–88	Fundus photography
Hsieh [15]	2017	China	Retrospective cohort study	Hospital-based	Median 6.6	761	T2DM	56.4	Mean 63.6	Ophthalmoscope FFA
Juutilainen [16]	2007	Finland	Prospective cohort study	Population-based	18	824	T2DM	51.6	45–64	Ophthalmoscope
Liew [17]	2008	Australia	Prospective cohort study	Population-based	12	199	T2DM	NA	NA	Fundus photography
Lövestam-Adrian [18]	2006	Sweden	Prospective cohort study	Hospital-based	10	363	T2DM	64.5	Mean 54.1	Fundus photography
Mottl [19]	2014	United States	Prospective cohort study	Hospital-based	Mean 5	3210	T2DM	61.8	Mean 61.3	Fundus photography
Ono [20]	2002	Japan	Retrospective cohort study	Hospital-based	Median 11.6	223	T2DM	77.1	Mean 60.3	Fundus photography FFA

DM: diabetes mellitus, DR: diabetic retinopathy, FFA: fundus fluorescein angiography, NA: not available

**Fig. 2:**
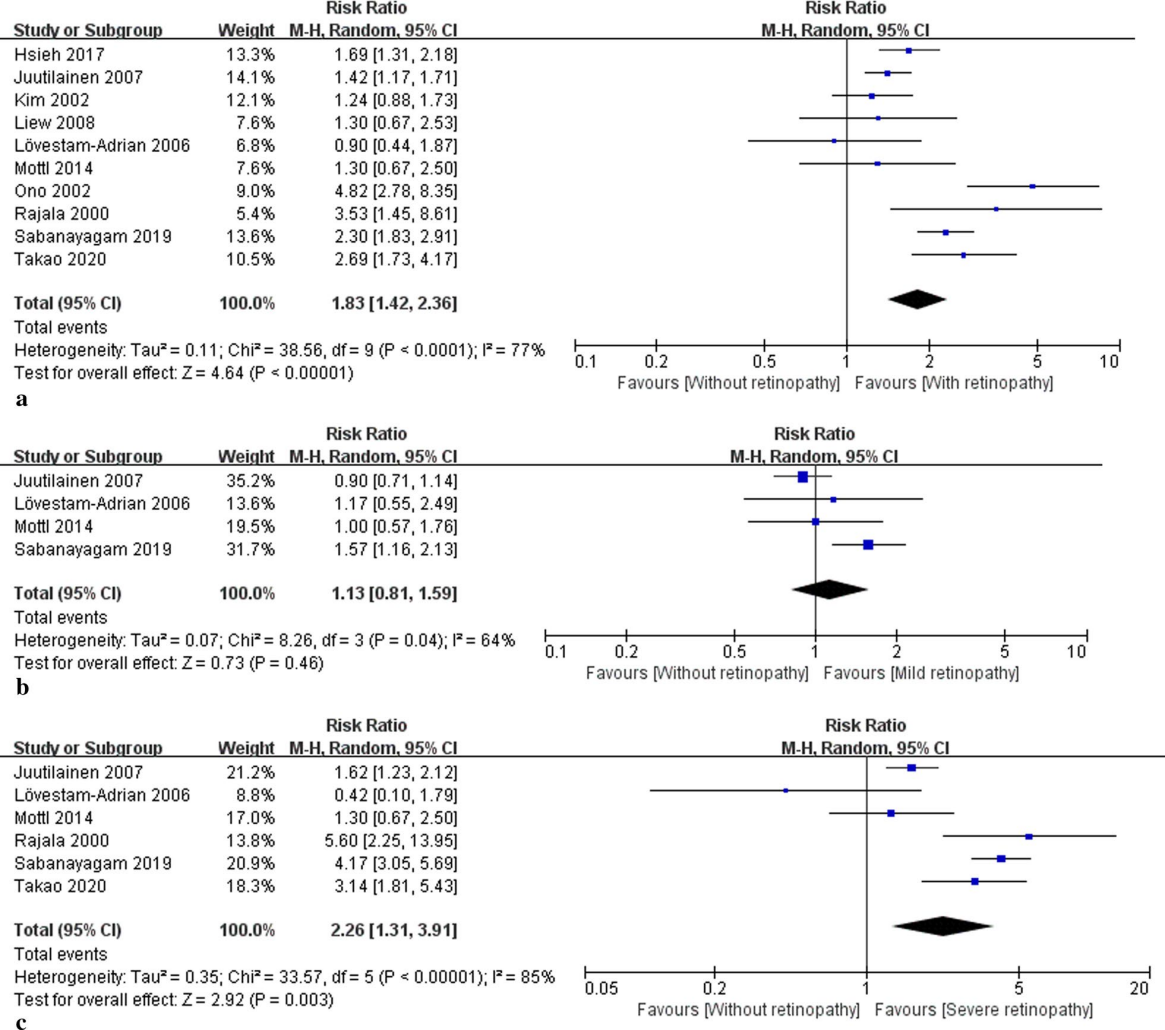
Forest plot showing the RRs of DR (**a**), mild DR (**b**) and severe DR (**c**) for CVD mortality. RR: risk ratio, DR: diabetic retinopathy, CVD: cardiovascular disease, CI: confidence interval

Overall, the pooled RR of DR, mild DR, and severe DR for CVD mortality was 1.83 (95% confidence interval (CI): 1.42, 2.36; *p* < 0.001; I^2^ = 77%), 1.13 (95% CI 0.81, 1.59; *p* = 0.46; I^2^ = 64%), and 2.26 (1.31, 3.91; *p* = 0.003; I^2^ = 85%), respectively, compared to those without DR (Fig. 2). In T2DM, the patients with DR had a significantly higher CVD mortality than those without (RR: 1.69; 95% CI 1.27, 2.24; *p* < 0.001; I^2^ = 75%).

Subgroup analysis was performed according to various regions, study design, data source, and follow-up period (Table 2). DR patients had significantly higher CVD mortality in both Asian (RR: 2.18; 95% CI 1.53, 3.11; *p* < 0.001; I^2^ = 82%) and western countries (RR: 1.46; 95% CI 1.22, 1.74; *p* < 0.001; I^2^ = 31%). In studies with more than 5-, 10- and 15-years follow-up, the RR of DR for CVD mortality was 1.86 (95% CI 1.40, 2.46;* p* < 0.001; I^2^ = 78%), 1.89 (95% CI 1.13, 3.16;* p* = 0.020; I^2^ = 84%), and 1.89 (95% CI 1.01, 3.56;* p* = 0.049; I^2^ = 86%), respectively. For those who underwent cardiovascular surgery, there was no significant correlation of DR and CVD mortality (RR: 2.40; 95% CI 0.63, 9.18; *p* = 0.200; I^2^ = 94%). Only 1 study analyzed the association of DR and CVD stratified by gender [16]. The results demonstrated the hazard ratio (HR) of CVD mortality in male was 1.30 (95% CI 0.86, 1.96) for mild retinopathy and 3.32 (95% CI 1.61, 6.78) for severe retinopathy. And the HR in female was 1.71 (95% CI 1.17, 2.51) for background retinopathy and was 3.17 (95% CI 1.38, 7.30) for severe retinopathy.

**Table 2:** Subgroup analysis showing the pooled RRs of DR for CVD mortality

Subgroups	Studies	Participants	RR (95% CI)	*P* value	Heterogeneity	
					I^2^, %	*P* value
Type 2 DM	8	7596	1.69 (1.27, 2.24)	< 0.001	75	< 0.001
Regions						
Asian countries	5	6017	2.18 (1.53, 3.11)	< 0.001	82	< 0.001
Western countries	5	4887	1.46 (1.22, 1.74)	< 0.001	31	0.22
Study design						
Prospective	7	8132	1.54 (1.16, 2.04)	0.003	70	0.003
Retrospective	3	2772	2.69 (1.49, 4.85)	0.001	84	0.002
Data source						
Population-based	3	3903	1.68 (1.13, 2.52)	0.010	82	0.004
Hospital-based	7	7001	1.93 (1.31, 2.84)	< 0.001	78	< 0.001
Follow-up period						
More than 5 years	8	10,111	1.86 (1.40, 2.46)	< 0.001	78	< 0.001
More than 10 years	5	3360	1.89 (1.13, 3.16)	0.020	84	< 0.001
More than 15 years	2	2712	1.89 (1.01, 3.56)	0.049	86	0.008
After cardiovascular surgery	2	588	2.40 (0.63, 9.18)	0.200	94	< 0.001

RR: risk ratio, DR: diabetic retinopathy, CVD: cardiovascular disease, DM: diabetic mellitus, CI: confidence interval

**Fig. 3:**
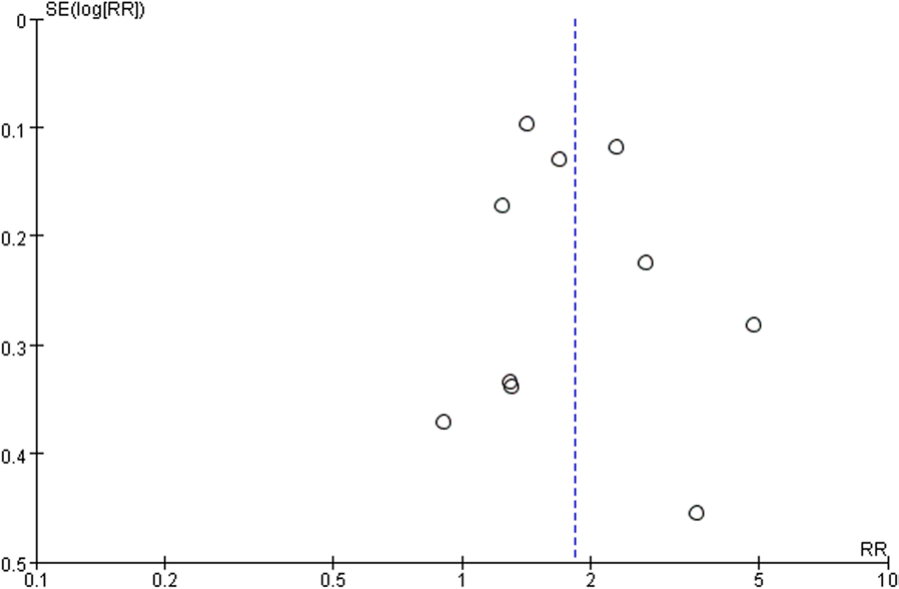
Funnel plot showing the test for publication bias of pooled RR of DR for CVD mortality. RR: risk ratio, DR: diabetic retinopathy, CVD: cardiovascular disease, SE: standard error

At last, Funnel plot was used to evaluate the publication bias of pooled RR of DR for CVD mortality (Fig. 3). No obvious publication bias was detected among the included studies in this meta-analysis.

## Discussion

This meta-analysis of cohort studies shows that the presence of any degree of DR is correlated with an increased risk for cardiovascular death in diabetes. Similar results are also obtained in subgroup analysis which is stratified by various regions, study design and follow-up period. These findings indicate the importance of early screening and management of diabetic patients with DR to reduce the mortality.

The present study finds diabetic patients with PDR or VTDR predict a doubled mortality of CVD than those without, but detects no significant association of mild DR and CVD mortality. A 7-year follow-up cohort study based on 1,059 non-insulin-dependent diabetes mellitus (NIDDM) also demonstrates CHD events (CHD death or nonfatal myocardial infarction) are only correlated with PDR instead of NPDR [21]. They assumed the correlation between DR and CHD may be due to similar pathophysiological backgrounds. Drinkwater et al. [22] also claimed the intensified CVD risk factor management should be considered for patients with at least moderate NPDR. However, the Age, Gene/Environment Susceptibility-Reykjavik Study (AGES-RS) explores the impact of retinopathy on mortality, and declares even minimal retinopathy is a significant predictor of increased mortality in older persons, irrespective of diabetes status [23]. Although it remains unclear whether mild DR will increase the mortality, it is also with significance to monitor NPDR patients since a part of them will finally develop to PDR.

In our study, DR is related with CVD mortality in studies with more than 10-year follow-up, which suggests DR serves as a valuable predictor for long-term survival rate in diabetic patients. A cohort study with 12-year follow-up also detects the presence of retinopathy is related to CVD death and myocardial infarction incidence in diabetes [24]. However, in the Central Australian Ocular Health Study, although diabetic patients with any DR have a higher all-cause mortality than those without, no relationship is detected in DR and cardiac mortality [25]. Therefore, further studies are still needed. In fact, assessing the mortality related to DM presents a lot of challenges. It cannot be precisely assessed from death certificates since deaths in diabetic patients usually result from one of its complications (e.g. stroke, heart disease and renal failure), which are regarded as the cause of death. Furthermore, it should be noticed that the assessment of long-term mortality risk in diabetes must include some related variables, such as glycemic control, glycosylated hemoglobin (HbA1c), renal function, blood pressure, dyslipidemia, and smoking habit [26].

The long-term mortality in diabetic patients with tractional retinal detachment (TRD) also deserves attention. Shukla et al. [27] compare the long-term all-cause mortality rate in TRD population and those diabetic patients with minimal to no retinopathy. They calculate a 48.7% 10-year all-cause mortality in TRD patients and even a higher mortality in those with worse vison. The results indicate TRD requiring vitrectomy surgery is a marker for poor long-term survival. Banerjee et al. [28] observe the mortality of 148 PDR patients undergoing vitrectomy surgery, and detect the 3-, 5- and 7-year survival rates are 94%, 86% and 77%, respectively. In an Australian population, the 5-, 7- and 9-year survival rates of diabetic patients undergoing vitrectomy for DR are 84.4%, 77.9% and 74.7%, respectively, with CVD as the most common cause of death [29].

It should be clarified that the main findings in our study cannot be applied in T1DM, since the majority of included participants were T2DM. The mortality and risk factors have been also evaluated in T1DM, and similar results were obtained. In an observational cohort study of 725 African-Americans with T1DM, the 3-year all-cause mortality is 18.1%, and 90% of the deceased are diagnosed with DR [30]. In a 12-year observation study of 462 T1DM, the relative risk for death is 7.0 times higher in patients with sight-threatening retinopathy, compared with those with no retinopathy [31]. However, this association disappears when retinopathy was adjusted for presence of macroalbuminuria. In addition, the 20-year mortality is calculated in a Germany cohort with T1DM, and the results also detect no association of DR and mortality [32]. Therefore, further studies are needed and various confounding factors should be taken into account.

The limitations of this study should be also noted. First, different definitions of CVD among the included investigations may influence the results. Second, some included investigations are retrospective cohort studies, and there is no age- and gender-matched study included in the meta-analysis. Third, some included studies contain a relatively small sample size. Fourth, the heterogeneity among the studies is large, though subgroup analysis is performed and random effects model is applied. Fifth, due to the limited data from the included studies, we fail to calculate the association of DR and specific cause of CVD death, such as CHD, arrhythmia, and sudden cardiac death. Sixth, we do not examine the contribution of individuals’ baseline characteristics (e.g. age, gender, HbA1c, and duration of DM) as well as therapeutic impacts in evaluating the relationship between DR and CVD mortality. Seventh, we do not evaluate the degree to which DR is itself a sign of poorly controlled CVD risk factors. And it is hard to prove whether DR is an independent risk factor for CVD, or DR has overlapped effects with other factors for CVD, including kidney disease, hyperglycemia and hypertension. Eighth, we do not perform a further sub-group analysis comparing the different diagnostic procedure of DR. In addition, since the majority of the included participants are T2DM, the findings in our study cannot be directly applied in T1DM.

## Conclusion

Our results show that DR is a risk marker of cardiovascular death and severe DR predicts a doubled mortality of CVD in diabetes. These findings indicate the importance of early identification and management of diabetic patients with DR to reduce the risk of death.

## Data Availability

All data used for this meta-analysis has been contained within the manuscript.

## Abbreviations

DM: Diabetes mellitus
IDF: International diabetes federation
CVD: Cardiovascular disease
DR: Diabetic retinopathy
NPDR: Non-proliferative diabetic retinopathy
PDR: Proliferative diabetic retinopathy
WESDR: Wisconsin epidemiologic study of diabetic retinopathy
RR: Risk ratio
CI: Confidence interval
VTDR: Vison-threatening diabetic retinopathy
CHD: Chronic heart disease
PCI: Percutaneous coronary intervention
CABG: Coronary artery bypass graft

## References

[1] Zimmet P, Alberti KG, Magliano DJ, Bennett PH (2016). Diabetes mellitus statistics on prevalence and mortality: facts and fallacies. Nat Rev Endocrinol.

[2] International Diabetes Federation. IDF Diabetes Atlas 7th edition. 2016. https://www.diabetesatlas.org. 10.1111/1753-0407.12453 27461234

[3] Zheng Y, Ley SH, Hu FB (2018). Global aetiology and epidemiology of type 2 diabetes mellitus and its complications. Nat Rev Endocrinol.

[4] Cheung N, Mitchell P, Wong TY (2010). Diabetic retinopathy. Lancet.

[5] Klein R. Epidemiology of diabetic retinopathy. In: Diabetic Retinopathy. Duh E, ed. Totowa: Humana Press. 2008.

[6] Varma R (2008). From a population to patients: the Wisconsin epidemiologic study of diabetic retinopathy. Ophthalmology.

[7] Klein R, Klein BE, Moss SE, Cruickshanks KJ (1999). Association of ocular disease and mortality in a diabetic population. Arch Ophthalmol.

[8] Klein BE, Klein R, McBride PE, Cruickshanks KJ, Palta M, Knudtson MD (2004). Cardiovascular disease, mortality, and retinal microvascular characteristics in type 1 diabetes: Wisconsin epidemiologic study of diabetic retinopathy. Arch Intern Med.

[9] Kramer CK, Rodrigues TC, Canani LH, Gross JL, Azevedo MJ (2011). Diabetic retinopathy predicts all-cause mortality and cardiovascular events in both type 1 and 2 diabetes: meta-analysis of observational studies. Diabetes Care.

[10] von Elm E, Altman DG, Egger M, Pocock SJ, Gøtzsche PC, Vandenbroucke JP; STROBE Initiative. The strengthening the reporting of observational studies in epidemiology (STROBE) statement: guidelines for reporting observational studies. PLoS Med. 2007; 4:e296. 10.1371/journal.pmed.0040296 PMC2020495 17941714

[11] Sabanayagam C, Chee ML, Banu R, Cheng CY, Lim SC, Tai ES (2019). Association of diabetic retinopathy and diabetic kidney disease with all-cause and cardiovascular mortality in a multiethnic Asian population. JAMA Netw Open.

[12] Takao T, Suka M, Yanagisawa H, Kasuga M. Combined effect of diabetic retinopathy and diabetic kidney disease on all-cause, cancer, vascular and non-cancer non-vascular mortality in patients with type 2 diabetes: A real-world longitudinal study. J Diabetes Investig. 2020. Epub ahead of print. 10.1111/jdi.13265 PMC7477514 32267626

[13] Kim YH, Hong MK, Song JM, Han KH, Kang DH, Song JK (2002). Diabetic retinopathy as a predictor of late clinical events following percutaneous coronary intervention. J Invasive Cardiol.

[14] Rajala U, Pajunpää H, Koskela P, Keinänen-Kiukaanniemi S (2000). High cardiovascular disease mortality in subjects with visual impairment caused by diabetic retinopathy. Diabetes Care.

[15] Hsieh YM, Lee WJ, Sheu WH, Li YH, Lin SY, Lee IT (2017). Inpatient screening for albuminuria and retinopathy to predict long-term mortality in type 2 diabetic patients: a retrospective cohort study. Diabetol Metab Syndr.

[16] Juutilainen A, Lehto S, Rönnemaa T, Pyörälä K, Laakso M (2007). Retinopathy predicts cardiovascular mortality in type 2 diabetic men and women. Diabetes Care.

[17] Liew G, Wong TY, Mitchell P, Cheung N, Wang JJ (2009). Retinopathy predicts coronary heart disease mortality. Heart.

[18] Lövestam-Adrian M, Hansson-Lundblad C, Torffvit O (2007). Sight-threatening retinopathy is associated with lower mortality in type 2 diabetic subjects: a 10-year observation study. Diabetes Res Clin Pract.

[19] Mottl AK, Pajewski N, Fonseca V, Ismail-Beigi F, Chew E, Ambrosius WT (2014). The degree of retinopathy is equally predictive for renal and macrovascular outcomes in the ACCORD Trial. J Diabetes Complications.

[20] Ono T, Kobayashi J, Sasako Y, Bando K, Tagusari O, Niwaya K, et al. The impact of diabetic retinopathy on long-term outcome following coronary artery bypass graft surgery. J Am Coll Cardiol. 2002; 40(3):428–36. 10.1016/s0735-1097(02)01983-6 12142107

[21] Miettinen H, Haffner SM, Lehto S, Rönnemaa T, Pyörälà K, Laakso M (1996). Retinopathy predicts coronary heart disease events in NIDDM patients. Diabetes Care.

[22] Drinkwater JJ, Davis TME, Hellbusch V, Turner AW, Bruce DG, Davis WA (2020). Retinopathy predicts stroke but not myocardial infarction in type 2 diabetes: the Fremantle Diabetes Study Phase II. Cardiovasc Diabetol.

[23] Fisher DE, Jonasson F, Klein R, Jonsson PV, Eiriksdottir G, Launer LJ (2016). Mortality in older persons with retinopathy and concomitant health conditions: the age. Gene/Environ Suscep Reykjavik Stud Ophthalmol.

[24] Fuller JH, Stevens LK, Wang SL (2001). Risk factors for cardiovascular mortality and morbidity: the WHO Mutinational Study of Vascular Disease in Diabetes. Diabetologia.

[25] Landers J, Liu E, Estevez J, Henderson T, Craig JE (2019). Presence of diabetic retinopathy is associated with worse 10-year mortality among Indigenous Australians in Central Australia: The Central Australian ocular health study. Clin Exp Ophthalmol.

[26] Grauslund J. Long-term mortality and retinopathy in type 1 diabetes. Acta Ophthalmol. 2010; 88 Thesis1:1–14. 10.1111/j.1755-3768.2010.01906.x 20500731

[27] Shukla SY, Hariprasad AS, Hariprasad SM (2017). Long-term mortality in diabetic patients with tractional retinal detachments. Ophthalmol Retina.

[28] Banerjee PJ, Moya R, Bunce C, Charteris DG, Yorston D, Wickham L (2016). Long-term survival rates of patients undergoing vitrectomy for proliferative diabetic retinopathy. Ophthalmic Epidemiol.

[29] Liu E, Estevez J, Kaidonis G, Hassall M, Phillips R, Raymond G (2019). Long-term survival rates of patients undergoing vitrectomy for diabetic retinopathy in an Australian population: a population-based audit. Clin Exp Ophthalmol.

[30] Roy M, Rendas-Baum R, Skurnick J (2006). Mortality in African-americans with type 1 diabetes: the New Jersey 725. Diabet Med.

[31] Torffvit O, Lövestam-Adrian M, Agardh E, Agardh CD (2005). Nephropathy, but not retinopathy, is associated with the development of heart disease in Type 1 diabetes: a 12-year observation study of 462 patients. Diabet Med.

[32] Heller T, Kloos C, Lehmann T, Schiel R, Lorkowski S, Wolf G (2018). Mortality and its causes in a german cohort with diabetes mellitus Type 1 after 20 years of follow-up: the JEVIN trial. Exp Clin Endocrinol Diabetes.

